# Anatomical Variation of Age-Related Changes in Vertebral Bone Marrow Composition Using Chemical Shift Encoding-Based Water–Fat Magnetic Resonance Imaging

**DOI:** 10.3389/fendo.2018.00141

**Published:** 2018-04-04

**Authors:** Thomas Baum, Alexander Rohrmeier, Jan Syväri, Maximilian N. Diefenbach, Daniela Franz, Michael Dieckmeyer, Andreas Scharr, Hans Hauner, Stefan Ruschke, Jan S. Kirschke, Dimitrios C. Karampinos

**Affiliations:** ^1^Department of Diagnostic and Interventional Neuroradiology, Klinikum rechts der Isar, Technical University of Munich, Munich, Germany; ^2^Department of Diagnostic and Interventional Radiology, Klinikum rechts der Isar, Technical University of Munich, Munich, Germany; ^3^Department of Nutritional Medicine, Klinikum rechts der Isar, Technical University of Munich, Munich, Germany

**Keywords:** bone marrow, magnetic resonance imaging, spine, osteoporosis, age distribution

## Abstract

Assessment of vertebral bone marrow composition has been proposed as imaging biomarker for osteoporosis, hematopoietic, and metabolic disorders. We investigated the anatomical variation of age-related changes of vertebral proton density fat fraction (PDFF) using chemical shift encoding-based water–fat magnetic resonance imaging (MRI). 156 healthy subjects were recruited (age range 20–29 years: 12/30 males/females; 30–39: 15/9; 40–49: 4/14; 50–59: 9/27; 60–69: 5/19; 70–79: 4/8). An eight-echo 3D spoiled gradient-echo sequence at 3T MRI was used for chemical shift-encoding based water–fat separation at the lumbar spine. Vertebral bodies of L1–L4 were manually segmented to extract PDFF values at each vertebral level. PDFF averaged over L1–L4 was significantly (*p* < 0.05) higher in males than females in the twenties (32.0 ± 8.0 vs. 27.2 ± 6.0%) and thirties (35.3 ± 6.7 vs. 27.3 ± 6.2%). With increasing age, females showed an accelerated fatty conversion of the bone marrow compared to men with no significant (*p* > 0.05) mean PDFF differences in the forties (32.4 ± 8.4 vs. 34.5 ± 6.8%) and fifties (42.0 ± 6.1 vs. 40.5 ± 9.7%). The accelerated conversion process continued resulting in greater mean PDFF values in females than males in the sixties (40.2 ± 6.9 vs. 48.8 ± 7.7%; *p* = 0.033) and seventies (43.9 ± 7.6 vs. 50.5 ± 8.2%; *p* = 0.208), though the latter did not reach statistical significance. Relative age-related PDFF change from the twenties to the seventies increased from 16.7% (L1) to 51.4% (L4) in males and 76.8% (L1) to 85.7% (L4) in females. An accelerated fatty conversion of bone marrow was observed in females with increasing age particularly evident after menopause. Relative age-related PDFF changes showed an anatomical variation with most pronounced changes at lower lumbar vertebral levels in both sexes.

## Introduction

The bone marrow as non-mineralized component of bone contributes to skeletal and systemic metabolism. Magnetic resonance spectroscopy (MRS) and chemical shift encoding-based water–fat magnetic resonance imaging (MRI) allow the quantitative assessment of the traditional bone marrow components, hematopoietic (red) and fatty (yellow) tissue (1). MR-based changes in bone marrow composition have been shown to be associated with different diseases including osteoporosis, disorders of the hematopoietic system, and metabolic disorders such as obesity and diabetes (2–7).

Bone mineral density (BMD), which is known to decrease in osteoporosis was reported to be inversely correlated with bone marrow fat fraction and positively with bone marrow unsaturation level (8–12). Therefore, assessment of vertebral bone marrow composition has been proposed as imaging biomarker for osteoporosis associated fracture risk prediction, particularly in subjects with diabetes where BMD measurements are limited in their ability to predict bone strength (13).

In patients with multiple myeloma, only those responding to treatment demonstrated a significant increase in vertebral bone marrow fat fraction (14). Furthermore, visceral adipose tissue and HbA1c levels were associated with vertebral bone marrow fat fraction in diabetic postmenopausal women (15). Bredella et al. reported that serum lipid levels positively correlated with bone marrow fat fraction in obese subjects (16). Insulin-like growth factor 1 was positively associated with vertebral bone marrow fat fraction (17). After sleeve gastrectomy, lumbar vertebral bone marrow fat fraction increased, while body weight and visceral adipose tissue decreased (18). Based on these findings, MR-based measurements of vertebral bone marrow composition were proposed as an advanced surrogate marker for hematopoietic and metabolic disorders.

Thus, reference values of the vertebral bone marrow fat fraction with regard to age and sex are needed, but were so far primarily obtained by using single-voxel MRS at a single lumbar vertebral level (19, 20). Compared to MRS, chemical shift encoding-based water–fat MRI allows spatially resolved assessment of bone marrow fat composition (1). A sex-independent increase of proton density fat fraction (PDFF) with age has been recently reported in children and a decrease of PDFF from the lumbar to the cervical spine has been reported in both young adults and children (21, 22). In children, the anatomical variation of PDFF from the lumbar to the cervical spine was shown to be age dependent. Therefore, the purpose of our study was to investigate the anatomical variation of age-related PDFF changes at the lumbar spine in male and female adults by using chemical shift encoding-based water–fat MRI.

## Materials and Methods

### Subjects

The study was approved by the local institutional committee for human research. All subjects gave written informed consent before participation in the study.

Healthy subjects older than 20 years of age were included in this study. Exclusion criteria were: history of pathological bone changes such as hematological or metabolic bone disorders aside from osteoporosis, history of diabetes, and contraindications for MR imaging. In total, 156 healthy subjects were recruited: age range 20–29 years (twenties): 12/30 males/females; 30–39 years (thirties): 15/9 males/females; 40–49 years (forties): 4/14 males/females; 50–59 years (fifties): 9/27 males/females; 60–69 years (sixties): 5/19 males/females; 70–79 years (seventies): 4/8 males/females.

### MR Imaging

All subjects underwent 3T MRI (Ingenia, Philips Healthcare, Best, The Netherlands). An eight-echo 3D spoiled gradient-echo sequence was used for chemical shift-encoding based water–fat separation at the lumbar spine using the built-in-the-table posterior coil elements (12-channel array). The sequence acquired the eight echoes in a single TR using non-flyback (bipolar) read-out gradients and the following imaging parameters: TR/TE1/ΔTE = 11/1.4/1.1 ms, FOV = 220 mm × 220 mm × 80 mm, acquisition matrix size = 124 × 121, acquisition voxel size = 1.8 mm × 1.8 mm × 4.0 mm, receiver bandwidth = 1,527 Hz/pixel, frequency direction = A/P (to minimize breathing artifacts), 1 average, scan time = 1 min and 17 s. A flip angle of 3° was used to minimize T1-bias effects (23, 24).

### Vertebral Bone Marrow Fat Quantification

**Figure 1:**
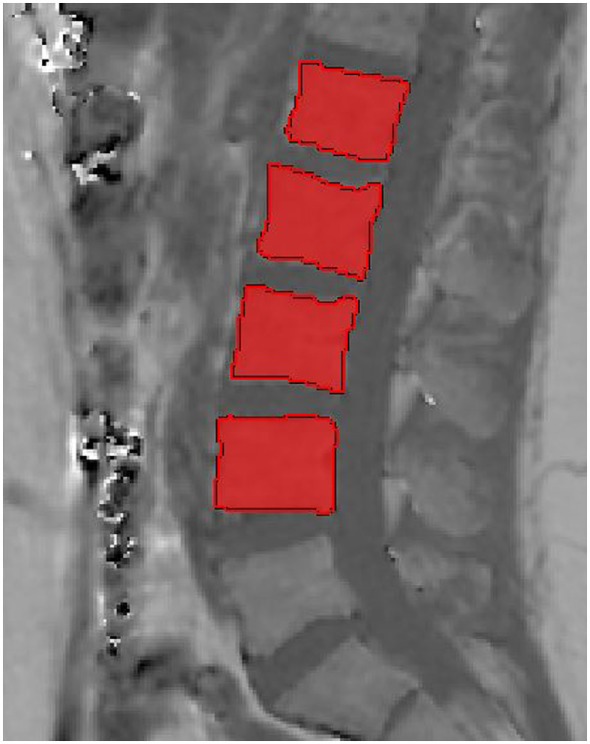
Representative segmentation of the vertebral bodies of L1–L4 in the proton density fat fraction map of a 29-year-old male.

The gradient echo imaging data were processed on-line using the fat quantification routine of the MR vendor. The routine procedure first performs a phase error correction and then a complex-based water–fat decomposition using a precalibrated seven-peak fat spectrum (25) and a single T_2_* to model the signal variation with echo time. The imaging-based PDFF map was computed as the ratio of the fat signal over the sum of fat and water signals. Similar to DXA measurements, the vertebral bodies L1–L4 were included in the analysis and manually segmented by a radiologist (Figure 1). The posterior elements and sclerotic changes of the endplates were excluded. Segmentation was performed on the PDFF maps by using the free open-source software Medical Imaging Interaction Toolkit, developed by the Division of Medical and Biological Informatics, German Cancer Research Center, Heidelberg, Germany; www.mitk.org. PDFF values were extracted at each vertebral level from L1 to L4. The MR image data and segmentations are not publicly available, but can be provided upon request.

### Statistical Analysis

The statistical analyses were performed with SPSS (SPSS Inc., Chicago, IL, USA). All tests were done using a two-sided 0.05 level of significance.

The Kolmogorov–Smirnov test indicated normally distributed PDFF values and non-normally distributed age and body mass index (BMI) values. Mean and SD of PDFF averaged over L1–L4, age, and BMI were computed for each age group, separately for males and females, and compared using *t*-tests and Wilcoxon–Mann–Whitney tests, respectively. Differences in PDFF of L1–L4 vertebral levels were determined by using paired *t*-tests. Relative age-related PDFF changes for both sexes were computed at each vertebral level as: (PDFF seventies − PDFF twenties)/PDFF twenties. PDFF changes through the five age groups were analyzed at each vertebral level, separately for males and females, by using analysis of variance.

## Results

Mean and SD of age and BMI for each age group, separately for males and females, are presented in Table 1.

**Table 1:** Number (*n*) of subjects (males/females) with mean and SD of age and body mass index (BMI) for each age group.

	Age (years)					BMI (kg/m^2^)				
	Male		Female			Male		Female		
	Mean	SD	Mean	SD	*p*-Value	Mean	SD	Mean	SD	*p*-Value
Twenties (*n* = 12/30)	26.6	1.8	24.8	2.5	**0.034**	25.7	3.5	23.7	3.6	0.129
Thirties (*n* = 15/9)	34.1	3.0	33.9	3.3	0.894	28.6	4.0	24.7	2.4	**0.013**
Forties (*n* = 4/14)	44.6	3.8	43.9	2.9	0.688	29.1	2.2	29.2	5.8	0.978
Fifties (*n* = 9/27)	52.8	2.0	55.8	3.0	**0.009**	30.4	7.0	27.5	5.4	0.193
Sixties (*n* = 5/19)	63.8	3.6	64.0	3.1	0.916	29.7	3.7	27.1	5.5	0.326
Seventies (*n* = 4/8)	74.5	1.3	74.7	3.2	0.931	26.4	4.1	24.6	6.5	0.632

*p-Values indicate differences between male and female subjects in the respective age group. Bold p-values are statistically significant (p < 0.05)*.

Proton density fat fraction averaged over L1–L4 was significantly (*p* < 0.05) higher in males than females in the twenties (32.0 ± 8.0 vs. 27.2 ± 6.0%) and thirties (35.3 ± 6.7 vs. 27.3 ± 6.2%). With increasing age, females showed an accelerated fatty conversion of the bone marrow compared to men with no significant (*p* > 0.05) mean PDFF differences in the forties (males: 32.4 ± 8.4%; females: 34.5 ± 6.8%) and fifties (males: 42.0 ± 6.1%; women: 40.5 ± 9.7%). The accelerated conversion process continued resulting in greater mean PDFF values in females compared to males in the sixties (males: 40.2 ± 6.9%; females: 48.8 ± 7.7%; *p* = 0.033) and seventies (males: 43.9 ± 7.6%; females: 50.5 ± 8.2%; *p* = 0.208), though the latter did not reach statistical significance (Table 2).

**Table 2:** Mean ± SD of proton density fat fraction (PDFF) averaged over L1–4 for each age group.

	Sex	Twenties	*p*-Value	Thirties	*p*-Value	Forties	*p*-Value	Fifties	*p*-Value	Sixties	*p*-Value	Seventies	*p*-Value
PDFF L1-4 (%)	Male	32.0 ± 8.0	**0.041**	35.3 ± 6.7	**0.008**	32.4 ± 8.4	0.607	42.0 ± 6.1	0.662	40.2 ± 6.9	**0.033**	43.9 ± 7.6	0.208
	Female	27.2 ± 6.0		27.3 ± 6.2		34.5 ± 6.8		40.5 ± 9.7		48.8 ± 7.7		50.5 ± 8.2	

*p-Values indicate differences between male and female subjects in the respective age group. Bold p-values were statistically significant (p < 0.05)*.

Proton density fat fraction increased from L1 to L4 in all subjects (L1: 35.9 ± 11.7%; L2: 36.2 ± 10.9%; L3: 37.2 ± 10.7%; L4: 39.5 ± 11.2% with L1 versus L2: *p* = 0.303; L2 versus L3: *p* < 0.001; L3 versus L4: *p* < 0.001).

**Figure 2:**
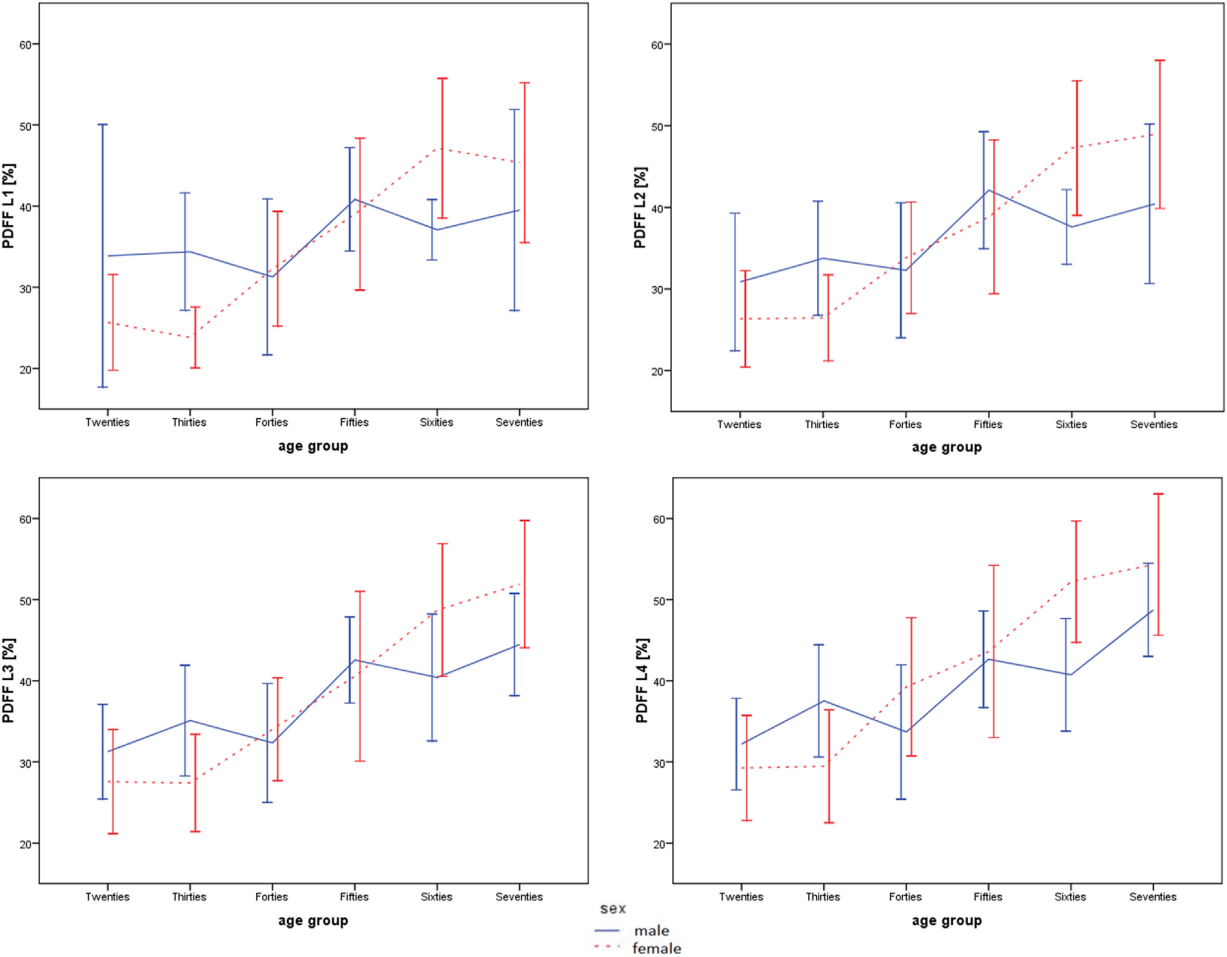
Mean and SD of proton density fat fraction (PDFF) values for males (blue/solid) and females (red/dashed) in L1–L4. Note the pronounced age-related fatty bone marrow conversion at lower lumbar vertebral levels.

**Figure 3:**
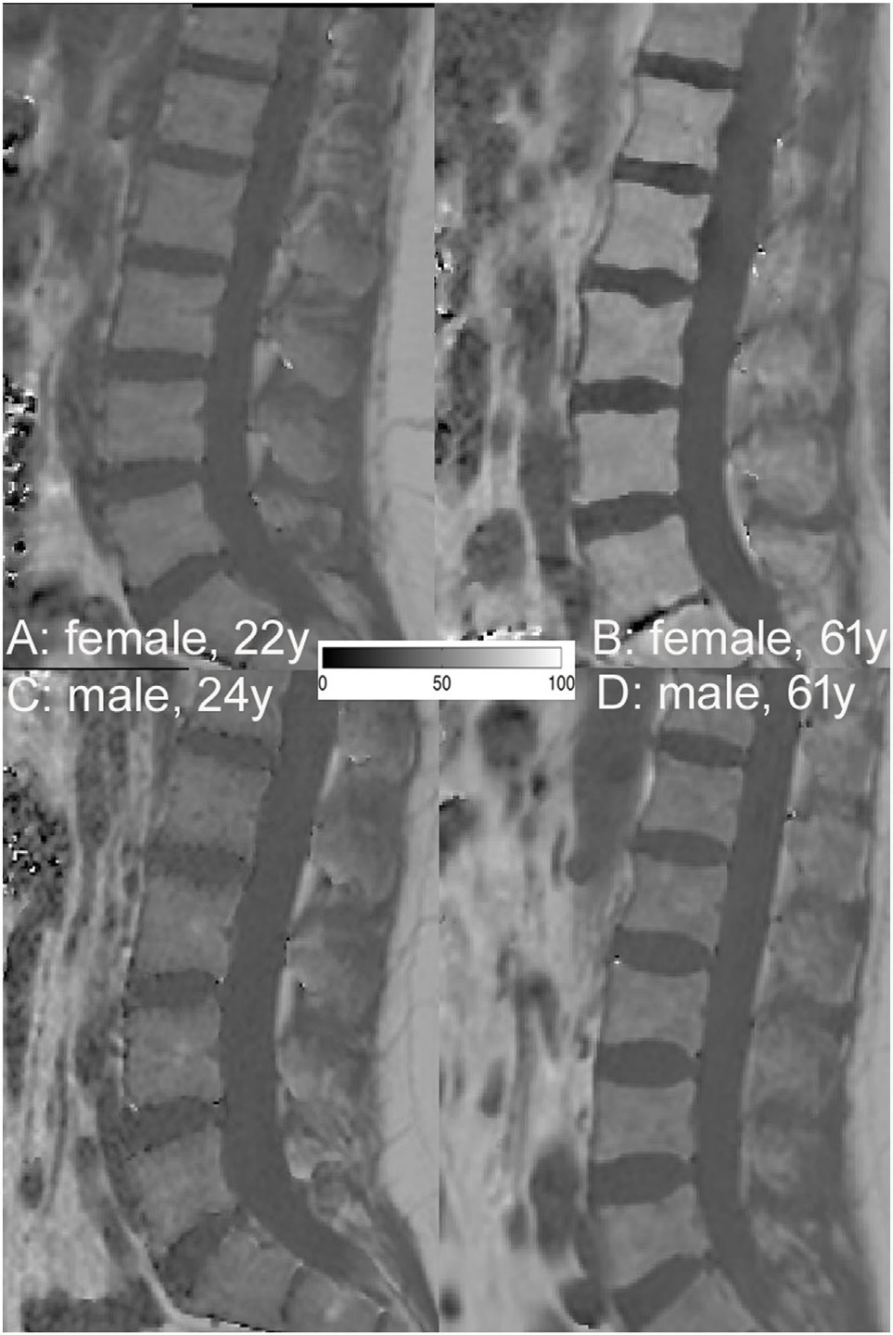
Representative proton density fat fraction (PDFF) maps of a 22- **(A)** and 61- **(B)** year-old female and a 24- **(C)** and 61- **(D)** year-old male subject, respectively. Note the lower PDFF values in A (mean PDFF: 26.2%) than in C (mean PDFF: 29.1%) and the greater PDFF values in **(B)** (mean PDFF: 62.1%) than in **(D)** (mean PDFF: 34.5%).

The age-related PDFF changes from the twenties to the seventies were dependent on the anatomical location and were most pronounced at lower lumbar vertebral levels in both sexes (Figures 2 and 3; Table 3). The relative age-related PDFF change from the twenties to the seventies increased from 16.7% (L1), 31.0% (L2), 42.3% (L3) to 51.4% (L4) in males and from 76.8% (L1), 85.8% (L2), 88.3% (L3) to 85.7% (L4) in females. The PDFF changes through the six age groups were significantly different (*p* < 0.05) at all vertebral levels in females and at L3 and L4 level in males.

**Table 3:** Anatomical variation of proton density fat fraction (PDFF) values for each age group and sex.

	PDFF L1 (%)				PDFF L2 (%)				PDFF L3 (%)				PDFF L4 (%)			
	Male		Female		Male		Female		Male		Female		Male		Female	
	Mean	SD	Mean	SD	Mean	SD	Mean	SD	Mean	SD	Mean	SD	Mean	SD	Mean	SD
Twenties	33.9	16.2	25.7	5.9	30.9	8.4	26.3	5.9	31.3	5.8	27.6	6.4	32.2	5.6	29.3	6.5
Thirties	34.4	7.2	23.8	3.8	33.8	7.0	26.4	5.3	35.1	6.8	27.4	6.0	37.5	6.9	29.5	7.0
Forties	40.8	22.8	32.3	7.1	41.3	21.4	33.8	6.8	35.6	9.7	34.0	6.3	35.1	7.9	39.3	8.5
Fifties	40.8	6.4	39.0	9.4	42.1	7.2	38.8	9.4	42.6	5.3	40.6	10.5	42.7	6.0	43.6	10.6
Sixties	37.1	3.7	47.1	8.6	37.,6	4.6	47.3	8.3	40.4	7.8	48.7	8.2	40.7	6.9	52.2	7.5
Seventies	39.5	12.4	45.4	9.9	40.4	9.8	48.9	9.1	44.5	6.3	51.9	7.8	48.7	5.7	54.3	8.7

Relative age-related PDFF change (%)	16.7 (*p* = 0.730)		76.8 (***p* < 0.001**)		31.0 (*p* = 0.100)		85.8 (***p* < 0.001**)		42.3 (***p* = 0.002**)		88.3 (***p* < 0.001**)		51.4 (***p* = 0.001**)		85.7 (***p* < 0.001**)	

*Relative age-related PDFF change was computed at each vertebral level as: (PDFF seventies − PDFF twenties)/PDFF twenties. Bold *p*-values indicate significantly (*p* < 0.05) different PDFF values through the five age groups at respective vertebral level*.

## Discussion

The central finding of our study was that vertebral PDFF assessed with chemical shift encoding-based water–fat MRI was dependent on age and sex in healthy adults. An accelerated fatty conversion of the bone marrow was observed in females as compared to males. Age-related PDFF changes from the twenties to the seventies showed an anatomical variation with most pronounced changes at lower lumbar vertebral levels in both sexes.

In 2001, Kugel et al. presented a study of 154 volunteers (age range: 11–95 years; 70 males, 84 females) who underwent single-voxel MRS at L3 level using a PRESS sequence (TR/TE 2,000/40 ms) (20). They reported lower relative fat signal intensity (corrected for T2 relaxation based on average T2 relaxation times) in females than males over all age groups. The relative fat signal intensity increased with age from 24% in the age group 11–20 years of age to 54% in the group greater than 61 years of age. Thus, an age- and sex-dependency of vertebral bone marrow fat was demonstrated. However, PDFF as determined in our study was not derived, which is an important technical limitation. Griffith et al. obtained single-voxel MRS at L3 level in 259 healthy subjects (age range: 62–90 years; 145 females, 114 males) also using PRESS (TR/TE 3,000/25 ms) (19). They specifically analyzed sex differences in vertebral bone marrow fat content in subjects aged 61–70, 71–80, and 81–90 years, which was not covered by Kugel et al. A considerable increase in vertebral bone marrow fat content was observed in females between 55 and 65 years of age resulting in greater vertebral marrow fat content in females compared to males older than 60 years of age (19). The main technical limitation of the study was the missing correction for MR relaxation effects, which confounds the fat quantification. We extracted vertebral bone marrow PDFF, which is advantageous as confounding factors including multiple peaks in the fat spectrum, T1-bias, and T2*-decay effects were taken into account (1). Water–fat imaging was previously validated against single-voxel MRS and good agreement for vertebral bone marrow PDFF measurements has been demonstrated (26).

Despite these technical issues and difficulties in comparing absolute bone marrow fat fraction values, our study results are in consistency with the single-voxel MRS studies demonstrating an accelerated fatty conversion of bone marrow in females compared to males from the forties onward. This finding may be explained by the physiological changes due to menopause. While each single-voxel MRS acquisition is limited to one vertebral level, chemical shift encoding-based water–fat MRI allows spatially resolved assessment of bone marrow fat composition. Thus, we were able to capture anatomical variation of relative age-related PDFF changes at the lumbar spine with a scan time of 1 min and 17 s. Kugel et al. reported a scan duration of 1 min 23 s for single-voxel MRS at L3 level (20). Whole spine coverage of water–fat imaging was previously obtained within 3 min (in children) and 10 min (in adults) (21, 22).

Our study revealed relative age-related PDFF changes from the twenties to the seventies with most pronounced changes at lower lumbar vertebral levels in both sexes. This finding is in line with a multidetector computed tomography study by Valentinitsch et al. (27). They investigated age-related local bone loss at the spine in healthy subjects and reported an initial bone loss at L5 level in the fifties cohort extending up to T10 level in the seventies cohort. Therefore, the anatomical variation of age-related bone loss and fatty bone marrow conversion has to be taken into account for local osteoporotic vertebral fracture risk assessment or treatment monitoring. Furthermore, our results suggest that a spatially resolved PDFF map might be preferable to single-voxel MRS-based vertebral PDFF measurements.

Our study has limitations, which have to be acknowledged. First, the sample size of subjects in some age groups was relatively small, particularly those aged 70–79 years (*n* = 12). Second, PDFF values were extracted at L1–L4 level according to DXA measurements. Future studies are needed to assess age-related changes of the bone marrow at the cervical and thoracic spine and to obtain corresponding PDFF reference data. Third, the menopausal status was not assessed in all female subjects. Thus, analysis of PDFF differences based on menopausal status was not possible and is a limitation of the study. Finally, acquisition of the lipid saturation index of the bone marrow is not possible with the used water–fat imaging technique. Given its performance as advanced imaging biomarker in the context of osteoporosis, reference data of the lipid saturation index by using MRS are desirable in the future (13).

In conclusion, an accelerated fatty conversion of bone marrow was observed in females with increasing age due to menopause. Relative age-related PDFF changes from the twenties to the seventies showed an anatomical variation with most pronounced changes at lower lumbar vertebral levels in both sexes. The results allow insights in physiological changes of the vertebral bone marrow composition and may serve as reference data. Due to the anatomical variation of age-related changes in vertebral bone marrow, a spatially resolved PDFF map is advantageous compared to single-voxel MRS-based PDFF measurements.

## Ethics Statement

This study was carried out in accordance with the recommendations of the Committee of Human Research, Faculty of Medicine, Technical University of Munich. The protocol was approved by the Committee of Human Research, Faculty of Medicine, Technical University of Munich. All subjects gave written informed consent in accordance with the Declaration of Helsinki.

## Author Contributions

TB: concept of study design, data acquisition, data post-processing, statistical analysis, and drafting of manuscript. AR and MD: data post-processing and critical revision of manuscript. JS: data acquisition, data post-processing, and critical revision of manuscript. DF: subject recruitment, data acquisition, and critical revision of manuscript. MD: data acquisition and critical revision of manuscript. AS: subject recruitment, data acquisition, and critical revision of manuscript. HH: concept of study design, subject recruitment, and critical revision of manuscript. SR: data post-processing and critical revision of manuscript. JK: concept of study design, statistical analysis, and critical revision of manuscript. DK: concept of study design, data acquisition, data post-processing, statistical analysis, and critical revision of manuscript.

## Disclaimer

The funders had no role in study design, data collection and analysis, decision to publish, or preparation of the manuscript.

## Conflict of Interest Statement

The authors declare that the research was conducted in the absence of any commercial or financial relationships that could be construed as a potential conflict of interest.
